# The Effect of Stress Management Programs on Physiological and Psychological Components of Stress: The Influence of Baseline Physiological State

**DOI:** 10.1007/s10484-021-09508-0

**Published:** 2021-05-12

**Authors:** Violette Hoareau, Christelle Godin, Frédéric Dutheil, Marion Trousselard

**Affiliations:** 1 CEA, LETI, MINATEC Campus, 38054 Grenoble, France; 2 Physiological and Psychosocial Stress, Université Clermont Auvergne, CNRS, LaPSCo, 34 Avenue Carnot, 63037 Clermont-Ferrand, France; 3 University Hospital of Clermont–Ferrand, CHU Clermont–Ferrand, Emergency Medicine, 63000 Clermont-Ferrand, France; 4 University Hospital of Clermont–Ferrand, CHU Clermont–Ferrand, Occupational and Environmental Medicine, WittyFit, 63000 Clermont-Ferrand, France; 5 Neurophysiology of Stress, Neuroscience and Operational Constraint Department, French Armed Forces, Biomedical Research Institute-IRBA, Brétigny-sur-Orge, France; 6 APEMAC/EPSAM, EA 4360, Ile du Saulcy, BP 30309, 57006 Metz Cedex 1, France; 7 French Military Health Service Academy, 1 Place Alphonse Laveran, 75230 Paris cedex 05, France

**Keywords:** Stress management programs, Chronic stress, Heart rate variability (HRV), Military fire-fighters

## Abstract

Military personnel are particularly exposed to stressful events, and overexposure to stress is both physically and mentally unhealthy. While stress management programs, such as the Tactics of Optimized Potential (TOP) and Heart Coherence (HC) have been implemented, their efficiency remains to be evaluated. The objective of this randomized control trial was to evaluate the effectiveness of the two programs among a young male population of 180 military fire fighter recruits. Based on two psychological, and one physiological measurement, namely heart rate variability (HRV), we found that both TOP and HC programs significantly increased HRV. This is promising as we know that higher HRV is consistent with better health, in most cases. Moreover, the TOP program significantly reduced perceived stress and negative mood, unlike the HC program. Combining these results, we conclude that while both TOP and HC programs influence physiological measurements, only the TOP modifies psychological evaluations. Finally, we distinguished the effects of the programs on two samples characterized by their HRV level. For the low HRV group, both programs tended to increase their HRV level, while for the high HRV group neither program had a significant effect.

## Introduction

In the military context, stress is not only a risk factor for good mental, physical and biological functioning, but also a major limiting operational factor. In the 1990s, the French army addressed the problem by developing a cognitive and emotional stress management program named Tactics of Optimized Potential (TOP). This comprehensive “tool box” combines mental imagery, relaxation and breathing techniques and the program is led by a TOP instructor. The aim is to train soldiers to optimize their resources in order to best-meet their objectives, and recover as quickly and fully as possible when in stressful situations. Although the program was originally created for soldiers, it can be used by anyone who needs to manage stress (Perreaut-Pierre, 2016). For example, it has been widely adopted by air traffic controllers, with apparently good effects, although results have never been published.

We examine the effectiveness of the TOP program, along with the so-called Heart Coherence (HC) cardio biofeedback program. HC was created by the HeartMath Institute, an American research center in California. It consists of coupling and synchronizing the cardiac rhythm with the phases of respiration; R–R intervals are shortened during inspiration and lengthened during expiration. Deep, regular breathing has been found to increase heart rate fluctuation and respiratory sinus arrhythmia, and it appears capable of optimizing the balance between sympathetic and parasympathetic systems (Russo et al., 2017). The latter are the two components of the autonomous nervous system; they are also known as the fight-or-flight mechanism, and the relaxation response, respectively. Deep breathing is commonly used in various relaxation methods, such as qigong or yoga, but the beneficial effects on well-being remain to be disentangled (Zaccaro et al., 2018).

The main objective of our study was, therefore, to test and compare the efficiency of the two programs. The first step was to measure the level of chronic stress and its evolution, for each participant, before and after the training program. However, while the stress response can be evaluated by psychobiological mechanisms, there is no simple, validated, experimental stress assessment. In practice, it is a psychological construct, and can be summarized as an adaptive response to a perceived danger or threat that involves physiological, cognitive, affective, and behavioral components. Like any construct, there are several operational definitions that determine how to measure it. In the specific case of stress, the literature provides two main operational tools: psychological questionnaires and physiobiological measurements. Psychological questionnaires are subjective, while physiobiological measures are objective. Assessment methods based on questionnaires primarily focus on an individual’s subjective perception of stress, while physiobiological measures reflect a state we may be unaware of. As there is no gold standard test, an interesting approach is to combine complementary psychological and physiological measures, to examine the effectiveness of stress management programs. Thus, a secondary objective of our work was to test the correlation between different stress measurements, as it is reasonable to expect a correlation between different operational evaluations that both claim to measure the same construct.

Here, we report results for two psychological questionnaires, and one physiological measurement. The first questionnaire evaluated perceived stress, and the second perceived negative mood. Heart Rate Variability (HRV) was selected as the physiological measure, as it is considered to be an accurate method to assess stress. In humans, the time interval between heartbeats is not regular, and HRV captures this variation, which is modulated by the autonomic nervous system. In general, the term HRV refers to vagally mediated HRV indexed by time domain measures, such as the root mean square of successive differences (RMSSD), and frequency domain measures such as high-frequency HRV (Thayer & Lane, 2000, 2009). A higher HRV at rest is thought to indicate greater activity of the parasympathetic component, and vice versa. Thus, a fall in HRV indicates greater stress or, in other words, a less relaxed state of the body.

The use of this indicator has gained momentum in research because several studies have shown that low HRV may predict sudden cardiac death (see the review by Sessa et al., 2018). In healthy subjects, low RMSSD has been associated with impaired physiobiological recovery after laboratory stressors compared to high RMSSD (Weber et al., 2010). Several other studies have tested its potential as an indicator of the psychological stress response (Kim et al., 2018; Sin et al., 2016) and it is now clear that HRV is sensitive to psychological stress. However, it is also sensitive to other factors, such as age, medication, or the quality of sleep the previous night. Nevertheless, authors have highlighted that RMSSD is a relevant HRV marker for chronic stress risk, indicated by persistent ruminative thought processes (Carnaveli et al., 2018). Porges (1995) describe HRV as an index of not only chronic stress, but also vulnerability to stress. Specifically, the higher the RMSSD, the more the body and mind are resistant to a psychological or physical stressor. The latter observation raises the question of whether, if the stress response is a function of the physiological baseline state, the impact of a stress management program is a function of the baseline state? To answer this exploratory question, we tested the impact of the program on two groups: low and high HRV-RMSSD.

We begin by presenting the impact of the stress management program on stress measures for all participants. Then, we address differences in the impact of the program on the stress vulnerability of low and high HRV-RMSSD groups. Finally, we examine correlations between our different stress measurements.

## Materials and Methods

### Participants

Participants were newly-recruited, volunteer fire fighters (FFs) undertaking training at the French army’s training unit. The study was approved by the *Comité de Protection des Personnes sud-est VI* (France) (IDRCB: 2010-A00212-37). All participants were young men with: no endocrine disease; no recent extraprofessional life-stress events (such as death of a near relative, or divorce); no current illness; no use of medications to modulate inflammatory diseases (corticosteroids, anti-inflammatory drugs, immunomodulatory drugs); and no medications with chronotropic effects taken over the previous 6 months (β-blockers, diltiazem, verapamil, anxiolytics, or antidepressants).

The 180 recruits were randomly allocated to one of three groups of 60 people. It was estimated that, with 55 participants in each group, the study would have > 80% power to detect a clinically important between-group difference in perceived stress, assuming a mean between-group difference of 2 points from the control group, with a pooled standard deviation (SD) of 2.1 (on the basis of preliminary data), at an α level of 5%.

### Protocol

The two stress management programs began during the fourth month of FF training, and lasted two months. The end of the program coincided with a stressful event—the qualifying examination. Indeed, the training of these young recruits ends with a grading exam at the end of which they choose their assignment position. This exam took place the week before the end of the stress management program.

Both the TOP and HC programs involved structured training, with the same, experienced instructor. Each program consisted of two hours of stress management per week, for 8 weeks, with a short, daily practice task. In both cases, training was carried out in small groups of 5–10 FFs.

All three groups received a placebo drug, which they were told would help to regulate stress. Participants were given a box of pills, and instructed to take one every day. The control group only received the placebo, while TOP and HC groups received the placebo along with participation in one of the two programs.

Psychological (questionnaires) and physiological (HRV) measures were registered before (baseline) and after the stress management programs. HRV values were recorded by detecting heartbeats over time, using an ECG. The registration lasted five minutes, while FFs were seated in a quiet room.

### Stress Measurements

#### Psychological Measures

The first psychological measure was the Perceived Stress Scale) (Cerclé et al., 2008; Cohen et al., 1983). This index is composed of 14 items, rated on a 5-point Likert-type scale that ranges from “Never” to “Very often”. Participants were asked about their feelings and thoughts during the past month (e.g. “In the past month, how often have you been upset because of something that happened unexpectedly?”). Higher scores indicated higher perceived stress.

The second psychological measure was Negative Mood, extracted from the Profile of Mood States (Shacham et al., 1983). A checklist of 37 adjectives was presented to each participant, and he was asked to indicate how he had been feeling in the past week (including the day of the test). Feelings were evaluated on a 5-point scale that ranged from “Not at all” to “Extremely”. Six factors were then calculated: anger, confusion, depression, fatigue, tension and vigor. Negative Mood corresponded to the sum of these factors, except the vigor subscale. Higher scores indicated higher negative mood.

#### Physiological Measures

ECG signals were captured using the VITAPORT–II (TEMEC Instruments B.V., Kerkrade). This clinical and research device uses a 1000 Hz sampling rate to accurately detect R-wave peaks. HRV can be computed in many ways, starting from the R–R interval, which corresponds to the beat-to-beat interval of the instantaneous heart rate. From this basic information, temporal, frequential or non-linear analyses can be used to extract HRV information. For clarity, we chose to use only one feature: RMSSD. The benefits of using RMSSD include: (1) its resistance to the influence of breathing frequency, which is a problem in frequential measures (Penttilä et al., 2001) and (2) its ability to capture levels of parasympathetic activity over a short-term period.

A RMSSD score for each subject was calculated using KUBIOS HRV analysis software. The ECG was always performed between 14:00 and 18:00 to limit contextual variation.

### Statistical Analyses

We first tested the Gaussian distribution of the three dependent variables (Perceived Stress, Negative Mood and RMSSD) with the Shapiro–Wilk test. This found that Perceived Stress was normally distributed (p = 0.5), while both Negative Mood and RMSSD were not (both p < 0.001). Consequently, RMSSD and Negative Mood were log transformed.

The effects of the two programs were evaluated using an analysis of variance (ANOVA) on relative scores obtained by subtracting baseline scores from post-treatment scores. ANOVAs were run for each of the three dependent variables, independently. As our main objective was to test and compare the two programs, all pairwise comparisons were also evaluated with post hoc analyses. Cohen’s d was used to estimate effect sizes. Although Cohen (1988) states that d = 0.2, 0.5 and 0.8 correspond to small, medium and large effects, these values are simply conventions, and there is no straightforward way to interpret standardized effect sizes (https://rpsychologist.com/d3/cohend/).

In the next step, we used the median of the log-transformed RMSSD computed from the baseline period to subdivide each of the three groups into two subgroups (high and low HRV). To test the effects of the stress management program for each HRV group, ANOVAs were run on relative scores, which, as before, were obtained by subtracting baseline scores from post-treatment scores.

Correlations between measurements of stress were assessed using Pearson’s correlation coefficient. First, we tested the correlation between absolute values (both baseline and post-treatment scores). Using the same absolute values, we also tested for an effect of group using an ANOVA for the two psychological measures. Secondly, we tested the correlation between variation in scores. This was because it was possible that a lack of correlation for absolute values was due to high individual variation in self-reported, estimated stress. If we consider that, for each individual, self-reported estimates of stress are robust over time, it is more relevant to study the correlation between variation in each of the dependent variables.

Effects of the groups’ programs were evaluated using ANOVAs on the changes in outcome variables. Change scores were calculated as change by sub-tracting baseline (M4) scores from scores at the end of the programs (M6). This was done for each outcome variable separately.

#### Comparisons Between CBF and TOP Programs

Effects of the groups’ programs were evaluated using ANOVAs on the changes in outcome variables. Change scores were calculated as change by subtracting baseline (M4) scores from scores at the end of the programs (M6). This was done for each outcome variable separately. Comparisons between CBF and TOP programs.

Significance was set at p < 0.05. All statistical procedures were performed using python.

## Results

### Participants

Fourteen FFs were excluded due to traumatic injuries that occurred just before the beginning of the study. Of the 166 remaining subjects, another 33 were excluded either because they did not fully complete one of the questionnaires, or because of a poor-quality ECG. In a final step, we identified outliers. This was done by calculating the Z-score for variation (post minus pre) in each dependent variable. Conventionally, a Z-score ± 3 is considered to be an outlier. This only identified one subject, who was then excluded. Despite these exclusions, the composition of the three groups stayed relatively well-balanced: 40 subjects in the Control group, 44 in the HC group, and 48 in the TOP group.

### Stress Management Program Effect

The first finding was that no difference was detected between the three groups for the three dependent variables at the beginning of the experiment. This confirmed that randomization was effective. Statistics for RMSSD, Perceived Stress and Negative Mood were, respectively: p = 0.5, p = 0.7 and p = 0.8.

**Fig. 1:**
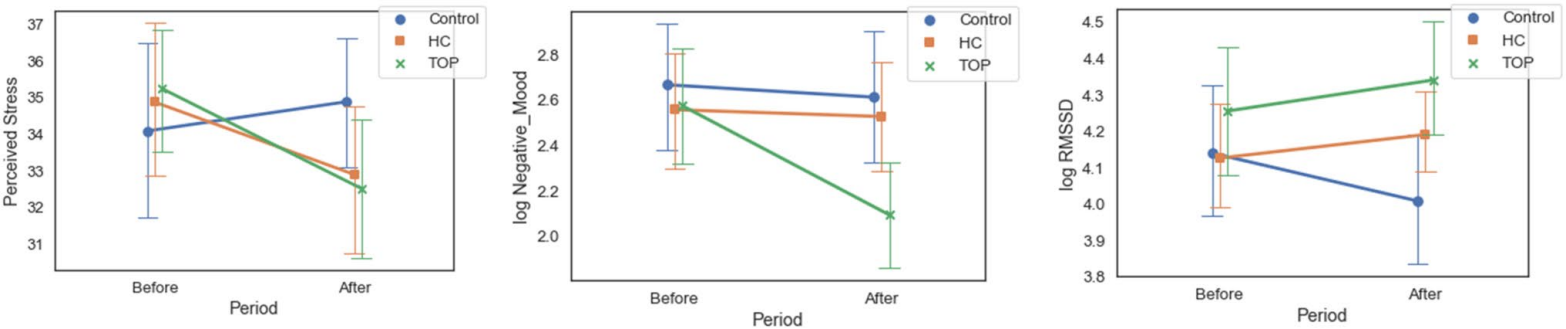
Mean and confidence interval for each of the three groups, for each period, and for each dependent variable. From left to right: Perceived Stress, log Negative Mood and log RMSSD

Post-treatment, Perceived Stress decreased among the groups, but did not reach significance [F (2, 129) = 3; p = 0.053] (Fig. 1, left). With respect to pairwise comparisons, the decrease in stress was significantly higher in the TOP group than the Control group [F (1, 86) = 5.6; p = 0.02; d =  − 0.5]. Stress also fell more in the HC group than the Control group, but the result was not significant [F (1, 82) = 2.9; p = 0.09; d =  − 0.37]. No difference was observed between the TOP and the HC group [F (1, 90) < 1; p = 0.6].

Change in log Negative Mood differed significantly between the three groups [F (2, 129) = 4.7; p = 0.01] (Fig. 1, middle). No difference was observed between the HC and the Control group [F (1, 82) < 1; p = 0.88]. However, values for the TOP group were significantly lower than for both the Control group [F (1, 86) = 5.8; p = 0.02; d =  − 0.52] and the HC group [F (1, 90) = 7.6; p = 0.007; d =  − 0.58].

Change in log RMSSD also differed significantly among the groups [F (2, 129) = 4; p = 0.02] (Fig. 1, right). Values for both HC and TOP groups were significantly higher than the Control group [F (1, 82) = 5.4; p = 0.02; d = 0.51 and F (1, 86) = 6.8; p = 0.01; d = 0.56 respectively]. No difference was observed between the TOP and the HC group [F (1, 90) < 1; p = 0.8].

Figure 1 illustrates that the TOP program had an effect on both psychological and physiological measures of stress, while the effect of the HC program is less clear, as the only significant difference was found for the physiological measure.

### Stress Management and Baseline HRV

**Fig. 2:**
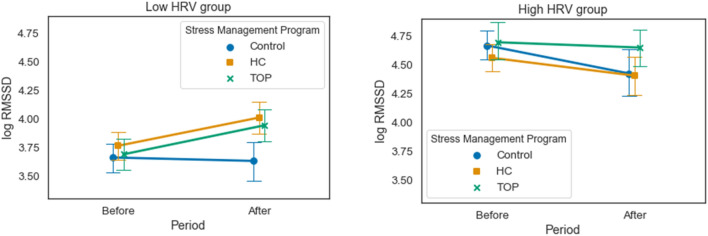
Mean and confidence interval of log RMSSD for each period, program, and HRV group. Left: results for the low HRV group; Right: results for the high HRV group

We noted that in cases where RMSSD was initially high (the high HRV group), it decreased following the HC program (p = 0.09), but not following the TOP program. On the other hand, if RMSSD was low at baseline (the low HRV group), it did not change after the program for the Control group, whereas there was a significant increase in both HC and TOP groups (p = 0.01, d = 0.8; p = 0.012, d = 0.8; respectively). Figure 2 illustrates the mean and confidence interval of log RMSSD for each period, each program, and each HRV group.

Concerning Perceived Stress and log Negative Mood pre- and post-treatment, no effect of HRV group was observed [F (1, 130) = 1.6; p = 0.24 and F (1, 130) < 1; p = 0.95, respectively].

### Correlation Between Stress Measurements

#### Absolute Values

**Fig. 3:**
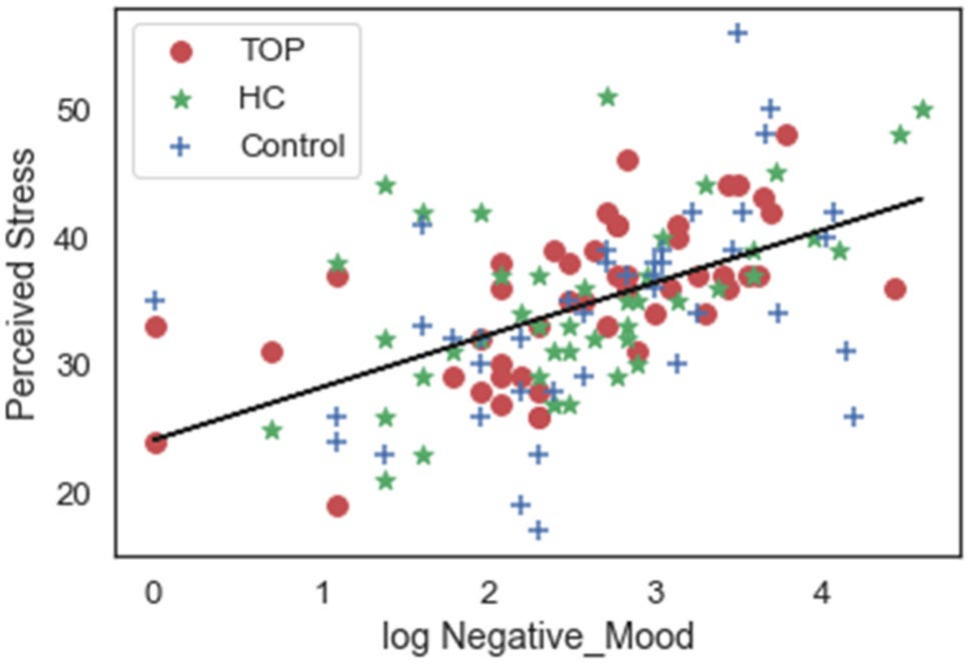
Correlation between the two psychological measures. The higher the Perceived Stress, the greater the Negative Mood

No correlation was observed between the physiological measure (RMSSD) and the two psychological measures (Perceived Stress and log Negative Mood). Moreover, we did not observe an effect of HRV group for the two psychological measures at baseline: Perceived Stress [F (1, 262) = 2.1; p = 0.15]; log Negative Mood [F (1, 262) = 1.8; p = 0.17]. However, Perceived Stress and Negative Mood were strongly correlated (r = 0.53; p < 0.001, Fig. 3).

#### Variation

**Fig. 4:**
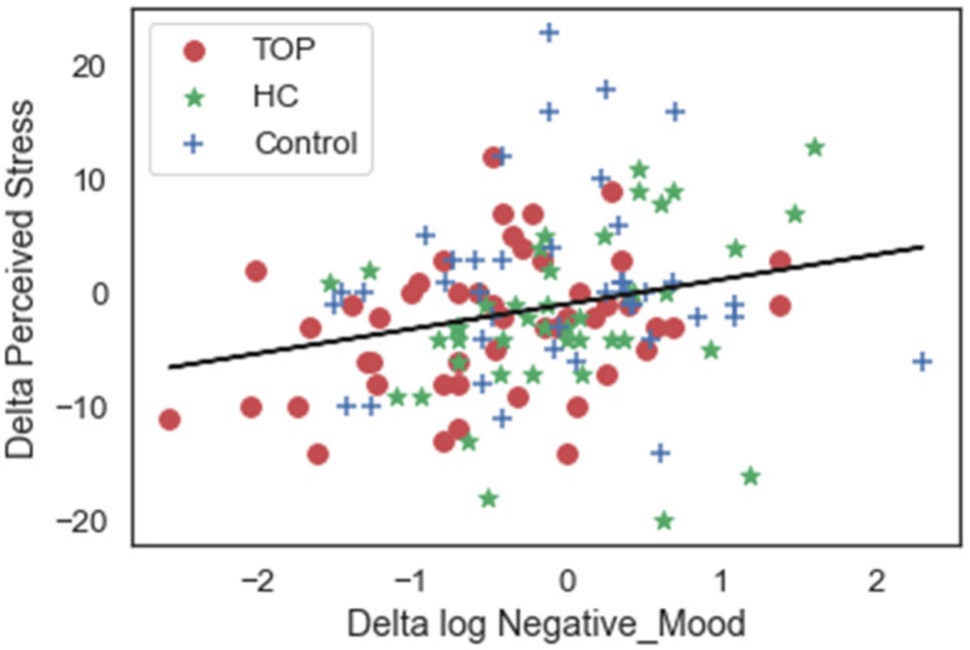
Correlation between variation in the two psychological measures. The more Perceived Stress increased, the more Negative Mood increased

We also tested the correlation between variation (post-minus pre-program) in the three dependent variables. Here again, no correlation was observed between physiological and psychological changes (r = 0.08; p = 0.34, for Perceived Stress and r = − 0.10; p = 0.2 for Negative Mood). However, as expected, there was a positive correlation between variation in the two psychological measures (r = 0.25; p = 0.004) (Fig. 4).

## Discussion

The purpose of this study was to evaluate and compare two stress management programs (TOP and HC) among a population of young FFs exposed to professional and academic stressors, as a function of their physiological baseline state. Although both programs aim to provide tools that enable people to cope with negative emotions and day-to-day stress, in order to limit physiological reactions that are harmful for health, they differ with respect to the target of the intervention. Our results are promising. First, we observed that HRV decreased less when FFs followed a stress management program (either HC or TOP). If we consider that higher HRV indicates higher parasympathetic activity, then both programs are beneficial, and could limit the harmful effects of stress. Secondly, we found that the TOP intervention limited subjective perceived stress and negative mood—but the HC program did not have the same effect. This pilot study seems to show that the TOP program is more effective, and has more global impact than the HC program. These results are logical, given that the HC program aims to act on physiology, while the TOP program aims to act on stress, via cognitive methods. In the case of the HC program, it is interesting to note that the subject is not aware of a physiological improvement, while the TOP approach seems to have more conscious impacts. This observation leads us to consider whether the two programs could complement each other or, possibly, it is best to begin with a HC program before launching a more complex program such as TOP.

Turning to the impact of the two programs with respect to baseline HRV levels, our results showed that both programs were beneficial, but especially for the low HRV group. For this group, both increased RMSSD, despite a stressful exam period. However, for the high HRV group, only the TOP program was effective in maintaining high HRV levels. For the Control group, baseline HRV was associated with opposite results after two months of taking a daily placebo. RMSSD did not fall in the low baseline HRV group, and it decreased in the high baseline group. It may be that for this group, there is a placebo effect that maintains the RMSSD level despite the stressful environment.

To sum up, both programs resulted in an increase in RMSSD for the low HRV group, but only the TOP program was effective (decreased perceived stress and negative mood) for the high HRV group. Low baseline RMSSD is considered to be a biomarker of stress vulnerability (Porges, 1995; Weber et al., 2010). For the low HRV group, interestingly, this biomarker appears to be sensitive to a stress management program, although RMSSD levels do not reach those of the high HRV group. Overall, these results seem to support the relevance of the two programs, especially for the low HRV group, who are the ones who need most help.

Concerning the impact of the program on psychological measures, no effect was observed for two the HRV levels. This result is understandable because neither of these two psychological variables correlates with the physiological variable, as we will discuss later.

Although our results are promising, it should be noted that the effect size is medium, with a Cohen’s d around 0.5. This indicates that a significant improvement would be seen in approximately 70% of the group that followed the program, compared to overall average variation in the Control group. Moreover, 80% of the two groups overlap, and there is a 64% chance that a randomly selected person who followed the treatment would score higher (better) than a randomly selected person in the Control group (see https://rpsychologist.com/d3/cohend/). It appears that these stress programs are not revolutionary, and that their impact will depend on the subject. At the same time, it seems that there is a general, beneficial impact. Furthermore, when considering these medium effect sizes, we should keep in mind that subjects did not (although they should have), practice regularly. It is reasonable to think that a regular practice would have had greater benefits.

Throughout this study, we used both physiological and psychological measures. We took the opportunity to test the correlation between these different types of stress measures, before and after the programs were run. As expected, we found a strong correlation between the two psychological measures: higher perceived stress is accompanied by higher negative mood at both baseline and post-treatment. However, no correlation was found between either psychological measure and HRV. These results highlight a discordance between subjective stress and physiological HRV improvements. At this point in time, few studies have examined links between psychological and physiological measures, and it appears that there is no strong evidence to link psychological and physiological states. Campbell and Ehlert (2012) showed that in only 25% of 49 studies there was a correlation between cortisol and scores of perceived stress, within the framework of an acute laboratory stress task. Sin et al. (2016) found a link between HRV and various subjective scores, but although they sampled a large number of participants (N = 909) the correlation was very weak.

This lack of a link between physiological and psychological measures seems to indicate that these variables do not capture exactly the same construct (chronic stress). In fact, the definition and measurement of chronic stress differs widely, and the problem is complex as stress is experienced on multiple levels: social, psychological and physiological. The multilevel in stress responses may explained the lack of consistency and thoroughness in measurements results (Epel et al., 2018). Indeed, it is necessary to take into account that self-reported stress measures and biological stress outcomes imply two different scale for assessments: self-questionnaires use limited Likert-type scaling including interval responses whereas biological outcomes are continu and most often not linear (Epel et al., 2018). The latter observation highlights a more global question: Did we really capture variation in stress? This limitation is found in all studies that measure “stress”. For example, HRV is strongly dependent on the context. Both pre- and post-program, we only measured ECG once. Therefore, observed variation in the HRV measure could be due to factors other than stress. In order to limit contextual variation, a future study could record the conditions (position, timing, etc.) of HRV recordings taken on each day of the experiment. This would provide continuous measurements for the whole month of training, and would be more robust to noise. Another avenue is to measure flexibility in vagal tone, in addition to the noisier, rest period measure. One way to measure this is to test physiological reactivity and the time necessary to return to calm in a stress test, followed by a rest period.

Another limitation of our study is the relevance of the placebo. We cannot be sure that giving all three groups a pill truly mitigated the placebo effect. Between-group comparisons of daily placebo intake were not run.

Concerning the protocol, the competitive exam for the choice of his assignment position was scheduled by the fire fighter chiefs just before the last stress management evaluation. It can be considered that this exam is a stressful challenge as it is suggested to the literature about students ‘exams (Roome & Soan, 2019). Nevertheless, we did not assess how the firefighters perceived this exam in terms of stress intensity and whether the stress management programs helped them to cope with the stress of the exam.

Finally, the duration of any benefits was not evaluated. Further studies need to include long-term follow-up to validate the real-life benefits of these programs, especially for subjects with a low baseline HRV.

Our results suggest some next steps for stress management. First, a combination of the two programs appears to be relevant. The HC program is simple and easy to execute, and it could be practiced each day, as a bottom-up action. The TOP program is more complex, and could be practiced each week, as a top-down action. Second, throughout the training period, participants should be equipped with a personalized tracking device, in the form of a watch that measures HRV, in order to customize the stress management program. This customization could take the form of quick, daily stress evaluations on a smartphone application, together with advice on appropriate exercises. Thirdly, a program focused on mind–body functioning could be added to improve introspection and increase awareness of physiological sensations. This focus should help to reduce the discordance between physiological and psychological states.

To conclude, despite certain limitations, our results are very promising, and indicate the benefits of stress management programs as a function of baseline HRV. They emphasize that stress evaluation needs to combine physiological and psychological assessments. As stress is a disease that affects both brain and body, the two approaches appear to be a relevant way to increase our capacity to act on day-to-day stress, and suggest some next steps for stress management.
